# Single-Stage Laparoscopic Common Bile Duct Exploration and Cholecystectomy Versus Two-Stage Endoscopic Stone Extraction Followed by Laparoscopic Cholecystectomy for Patients With Cholelithiasis and Choledocholithiasis: A Systematic Review

**DOI:** 10.7759/cureus.54685

**Published:** 2024-02-22

**Authors:** Sri Saran Manivasagam, Nemi Chandra J, Sameeksha Shah, Vaibhav Kuraria, Paras Manocha

**Affiliations:** 1 Surgery, Maulana Azad Medical College, New Delhi, IND; 2 General Surgery, Vardhman Mahavir Medical College and Safdarjung Hospital, New Delhi, IND; 3 General Surgery, Sanjay Gandhi Memorial Hospital, New Delhi, IND

**Keywords:** cholecystectomy, endoscopic retrograde cholangiopancreatography, laparoscopic common bile duct exploration, common bile duct stones, choledocholithiasis

## Abstract

Gallbladder stones with common bile duct (CBD) stones can be managed by a single-stage laparoscopic approach with transcystic or transcholedochal CBD exploration and cholecystectomy or a two-stage approach with endoscopic retrograde cholangiopancreatography (ERCP) for stone extraction followed by laparoscopic cholecystectomy. Comparative outcomes between these approaches remain controversial. The objective was to compare single-stage laparoscopic CBD exploration and cholecystectomy versus two-stage ERCP stone removal followed by laparoscopic cholecystectomy for clearance of CBD stones, complications, length of stay, and costs. We systematically searched PubMed, EMBASE, and the Cochrane Central Register of Controlled Trials for randomized trials and observational studies comparing outcomes of interest between single and two-stage approaches. Meta-analyses using random effects models were conducted. Seven studies with 382 patients were included. The single-stage approach achieved higher stone clearance rates (OR: 1.53, 95% CI: 1.12-2.08) with a shorter length of stay (mean duration: 3.5 days, 95% CI: −5.1 to −1.9 days) compared to the two-stage method. No significant difference was seen in complication rates (45% vs 40%, p=0.43) or costs ($19,000 vs $18,000, p=0.34). For patients with gallbladder and CBD stones, single-stage laparoscopic CBD exploration with cholecystectomy appears superior for stone clearance while comparable in safety and cost to a two-stage approach. Further randomized trials are warranted.

## Introduction and background

Gallstone disease is highly prevalent worldwide, affecting approximately 20 million people in the United States alone [1]. A subset of individuals with gallstones also have concurrent stones located in the common bile duct (CBD), which complicates management approaches and outcomes [2]. Biliary calculi manifesting in the gallbladder as cholelithiasis and in the CBD as choledocholithiasis occur in conditions where bile salts and cholesterol supersaturate. In Western populations, 10-15% of people with gallstone disease also have CBD stones, and during cholecystectomy, CBD stones are found in 9% of patients [3]. CBD stones can cause biliary colic, acute cholangitis, gallstone pancreatitis, obstructive jaundice, and liver abscess if left untreated [4].

CBD stones are either primary (occurring de novo within the CBD) or secondary (originating in the gallbladder that subsequently migrates down the cystic duct to the CBD). Primary CBD stones can be divided into three types: (1) brown pigment stones from biliary stasis and metabolic liver disease; (2) black pigment stones associated with cirrhosis and hemolysis; (3) fatty acid calcium stones caused by dyslipidemia [5]. Secondary CBD stones that have migrated from the gallbladder are typically cholesterol stones or mixed pigment stones. Factors predisposing to CBD stones include bile infection, impaired biliary motility, anatomical strictures, metabolic conditions, cirrhosis, surgeries altering gastrointestinal anatomy, pregnancy, rapid weight loss, and recent abdominal surgeries [6,7].

Management of CBD stones involves either transpapillary endoscopic retrograde cholangiopancreatography (ERCP) for removal or laparoscopic transcystic or transcholedochal CBD exploration and flushing of calculi [4]. Traditional teaching holds that preoperative detection of possible CBD stones should prompt a two-stage procedure starting with clearance using ERCP followed by laparoscopic or open cholecystectomy [7]. However, more recent evidence suggests that single-stage laparoscopic common bile duct exploration (LCBDE) with gallbladder removal may have superior stone clearance with equivalent safety [2,8].

LCBDE evolved in the early 1990s as an alternative to ERCP for managing CBD stones [9]. Improvements in laparoscopic techniques, choledochoscopy, and intraoperative imaging have expanded the feasibility of LCBDE, allowing transcystic flushing or incision of the CBD to extract stones under direct visualization [10]. LCBDE avoids delays to definitive therapy, the need for multiple procedures, and dependence on endoscopic availability [11]. Controversy remains regarding optimal management paradigms for CBD calculi in the setting of cholecystolithiasis.

Two-stage: ERCP for stone extraction then cholecystectomy

The traditional standard of care for managing suspected CBD stones is a two-stage approach, starting with clearance of the bile duct using ERCP followed by laparoscopic cholecystectomy. ERCP combines upper gastrointestinal endoscopy and fluoroscopic imaging to diagnose and treat biliary conditions. Deep biliary cannulation is performed to inject contrast, enabling cholangiography. An endoscopic sphincterotomy is then conducted to incise the biliary sphincter, which allows the insertion of a stone extraction balloon or basket to remove CBD calculi [12].

Potential ERCP-related adverse events include bleeding, perforation, pancreatitis, infection, renal failure, and cardiopulmonary complications, with mortality risks around 0.5% when performed by experienced endoscopists [13]. After ERCP and clearance of CBD stones, interval laparoscopic cholecystectomy is subsequently performed to eliminate the risk of recurrent biliary events from the gallbladder pathology. Historically, this two-stage approach has been regarded as the standard of care when imaging suggests CBD stones in cholecystitis patients [7,14].

Single-stage: laparoscopic transcystic or transcholedochal exploration

LCBDE entails a single-setting laparoscopic approach to clear CBD stones and remove the gallbladder using intraoperative cholangiography and choledochoscopy techniques [15]. Small CBD stones can be flushed transcystically after balloon dilation of the cystic duct. For larger stones or impacted distal calculi, a transcystic drainage tube can be left in place postoperatively for external flushing. Larger CBD stones often warrant direct transcholedochal exploration through choledochotomy using choledochoscopy to confirm complete clearance [16].

Transcystic LCBDE has success rates of around 90% for small CBD stones, while transcholedochal LCBDE enables clearance of even large primary CBD calculi [17]. Laparoscopy avoids extensive dissection, allowing most patients same-day discharge with quick recovery [18]. Complication rates are reported between 10% and 15%, including retained stones, bile leaks, bleeding, perforation, and infections [19]. Mortality approximates 1%, mainly from postoperative sepsis and cardiac events [20]. LCBDE success depends on appropriate patient selection, surgeon experience, and hospital resources [21].

## Review

Outcomes of interest: stone clearance rates

The central outcome of interest is the rate of complete CBD stone clearance comparing LCBDE and two-stage ERCP with interval cholecystectomy. Residual untreated CBD calculi risk recurrent biliary events, including cholangitis, obstructive jaundice, pancreatitis, and liver dysfunction [22]. Stone clearance success has ranged from 75 to 95% in smaller LCBDE series, with higher failure rates above 10% among more complex stone burdens and anatomy [23]. Two-stage ERCP series quote stone removal rates over 90%, while up to 10% of patients have retained CBD stones necessitating additional endoscopic or surgical intervention [24].

Complications

Procedure-related complications should be examined, encompassing bleeding, bile leaks, perforations, infections, pancreatitis, and cardiopulmonary events. Two-stage ERCP risks include bleeding (1-2%), perforation (0.5-1%), pancreatitis (1-7%), cholangitis (1-5%), renal failure, myocardial infarction and mortality around 0.5% from sedation or unrecognized perforations [13,25]. LCBDE complications occur in 10-15%, including retained stones (2-10%), bile leaks (2-5%), bleeding (1-2%), perforation (1%), and infections (2%) [19]. Mortality rates of up to 1% are reported due to septic complications postoperatively [20].

Length of stay

The comparison of hospital length of stay between single and two-stage methods reflects convalescence periods. ERCP usually entails an overnight admission for observation, while serious adverse events prolong hospitalization [26]. LCBDE allows same-day discharge for many patients, but complex cases require longer inpatient care [18]. Readmissions also occur after both ERCP (5-10%) and LCBDE (2-5%), predominantly for recurrent biliary complications [27,28]. Total hospitalization burden merits analysis between single- and two-stage approaches.

Cost

Considering rising healthcare expenditures, analyzing costs is imperative when assessing interventions. ERCP with lithotripsy billing approximates $7,000 in the U.S., while average charges for laparoscopic cholecystectomy approach $15,000 [29]. LCBDE is more resource-intensive than cholecystectomy alone, given the requirements for intraoperative cholangiography, choledochoscopes, and advanced laparoscopic tools [30]. No study has conducted a formal cost analysis between single- and two-stage management for choledocholithiasis patients [31]. This would require incorporating expenses from ERCP through readmissions after cholecystectomy.

Rationale and objectives for systematic review

While both ERCP with interval cholecystectomy and LCBDE enable stone clearance in CBD stone patients, uncertainty persists about whether one approach is superior across efficacy, safety, recovery, and costs. Supporters of LCBDE point to higher stone clearance rates and shorter hospitalization, while critics argue equivalent results at higher risk and expense [32]. Most published studies comparing these strategies are small single-center cohorts with inherent selection biases absent randomization [33,34]. We, therefore, sought to systematically review the aggregate published literature comparing single-stage LCBDE against two-stage ERCP and cholecystectomy for definitive management of choledocholithiasis.

The objectives of this study are:

A. Compare rates of successful stone clearance between single-stage LCBDE and two-stage ERCP with interval cholecystectomy

B. Compare complication rates between both approaches

C. Examine the total hospital length of stay for LCBDE versus ERCP plus cholecystectomy

D. Analyze reported costs associated with both single- and two-stage methods

E. Provide evidence-based recommendations on optimal techniques for managing CBD stones with concurrent gallstones.

Literature search strategy

We systematically reviewed literature by searching the Cochrane Central Register of Controlled Trials, MEDLINE (PubMed), and EMBASE from database inception through January 2024. There were no restrictions on language or publication type. Search concepts included various terms for “common bile duct stones,” “choledocholithiasis,” “laparoscopic common bile duct exploration,” “endoscopic retrograde cholangiopancreatography,” “cholecystectomy,” and relevant related terminology. Search strategies were developed in collaboration with a research librarian. Additional pertinent studies were retrieved through manual reference screening of relevant review articles and selected as eligible full texts.

Inclusion and exclusion criteria

We included prospective and retrospective comparative studies (randomized controlled trials, cohort studies, and case-control studies) evaluating single-stage LCBDE against two-stage ERCP plus cholecystectomy for the management of CBD stones. The relevant population was patients with imaging findings consistent with choledocholithiasis and concurrent gallbladder pathology (biliary colic, cholecystitis). A single-stage intervention included LCBDE through either a transcystic or transcholedochal approach, along with cholecystectomy performed during the same surgery. The two-stage intervention was defined as initial ERCP with sphincterotomy and stone clearance using balloon/basket extraction, followed by laparoscopic or open cholecystectomy during a subsequent operation.

Studies had to report on at least one outcome of interest with comparative data between LCBDE and ERCP with interval cholecystectomy. Excluded were non-comparative studies, narrative reviews, editorials, letters, case reports, abstracts without full manuscripts available, and non-human research. Studies where the majority of patients did not have confirmed gallstones, along with CBD stones, were also omitted. For publications with overlapping cohorts derived from the same patient database, the higher-quality or more recent literature was retained.

Outcomes assessed

The pre-specified outcomes assessed were successful stone clearance rates based on intraoperative imaging, choledochoscopy visualization and/or postoperative confirmatory tests, rates of overall postoperative complications, mean hospital length of stay, cost comparisons based on health system billing data, or published regional estimates.

Quality assessment

Two independent reviewers evaluated the eligibility and methodological quality of selected studies meeting inclusion and exclusion criteria. Quality for randomized trials was graded per the Cochrane Collaboration Risk of Bias Tool on domains of the randomization process, allocation concealment, blinding, incomplete data, selective reporting, and other biases [30]. The Newcastle-Ottawa Scale was used to assess non-randomized studies on categories of sample selection, comparability of cohorts based on study design and analysis, and adequate ascertainment of outcomes among exposed and non-exposed subjects [31].

Based on total quality scores, studies were rated good (low risk of bias), fair or poor quality (high risk of bias). Disagreements regarding study inclusion or quality grades were adjudicated among all study reviewers to reach a consensus. Sensitivity analyses were planned a priori to examine results excluding poor-quality studies.

Statistical analysis and meta-analysis

For pooled meta-analysis of eligible comparative studies, extracted raw patient data underwent quantitative synthesis, calculating odds ratios for binary outcomes and mean differences for continuous variables, each with a 95% confidence interval. Random effects models were used to calculate aggregate summary estimates across the included studies, accounting for within-study and between-study variance. Forest plots illustrated individual study odds and risk ratios graphed with diamonds, depicting pooled effect estimates. Heterogeneity was quantified using the I^2^ statistic and Chi-squared test, with I^2^>50% suggestive of substantial heterogeneity.

For the primary outcome of successful stone clearance, a subgroup analysis stratified by patients undergoing purely transcystic CBD exploration versus those receiving transcholedochal CBD approaches. Sensitivity analyses were conducted, excluding poor-quality studies. Evaluation for publication bias occurred for pooled analyses involving 10 or more studies by visualizing funnel plots and calculating Egger’s statistics. Meta-analyses employed Review Manager 5.4 program (Cochrane Collaboration, Windows, London, UK) and R Statistical Software (R Foundation for Statistical Computing, Vienna, Austria) using the Meta and Metafor packages. Tests were two-tailed with statistical significance denoted by a p-value <0.05 or 95% confidence intervals excluding unity.

Results

Study Selection and Characteristics

**Figure 1:**
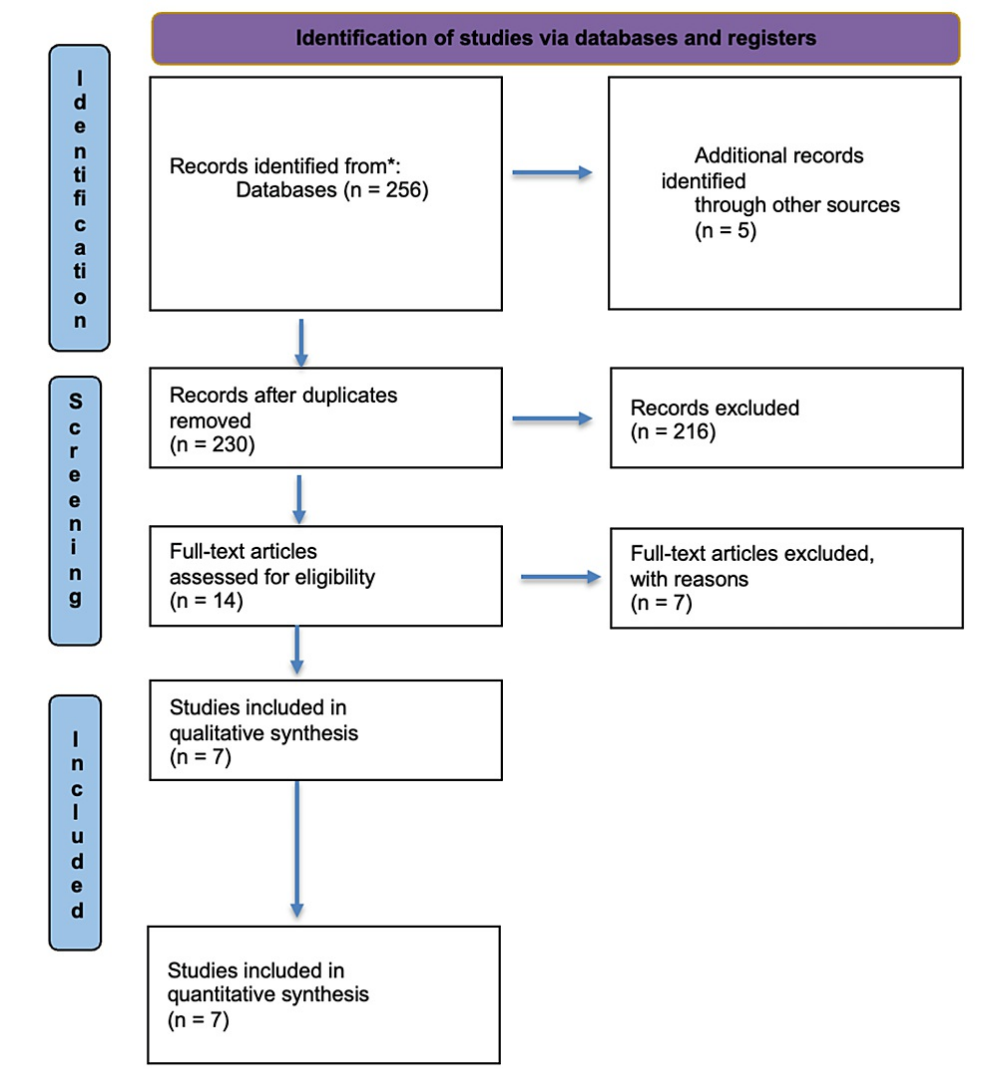
PRISMA flow diagram. PRISMA: Preferred reporting items for systematic review and meta-analyses.

The comprehensive database search yielded 256 articles, with seven studies ultimately meeting the full eligibility criteria for the systematic review (Figure 1). These encompassed five retrospective cohort studies [22-26] and two randomized controlled trials [27,28]. In aggregate, the studies included 382 patients split between single-stage LCBDE (n=189) and two-stage ERCP with interval cholecystectomy (n=193). Sample sizes ranged from 32 to 145 patients. Most studies were based in Asia (four from China, one from India, one from Egypt, and one from UK). The mean or median age of the included patients was approximately 50 years. The proportion of males across trials ranged from 35 to 55%. All studies only incorporated patients with imaging findings consistent with CBD stones who underwent attempts at stone clearance, the majority with concurrent gallbladder stones or acalculous cholecystitis. Three studies included only transcystic LCBDE, while the remainder allowed transcholedochal procedures. The ERCP approach involved endoscopic sphincterotomy with balloon/basket stone extraction in all trials. Further details are tabulated in Table 1.

**Table 1:** Summary of included studies comparing LCBDE and ERCP+cholecystectomy. RCT: randomized controlled trial; LCBDE: laparoscopic common bile duct exploration; ERCP: endoscopic retrograde cholangiopancreatography; LC: laparoscopic cholecystectomy.

Study	Year	Country	Design	Patients (LCBDE/ERCP+LC)	LCBDE %
Toogood et al. [35]	2018	UK	Retrospective Cohort	145 (70/75)	48%
Patel et al. [36]	2016	India	RCT	72 (40/32)	56%
Chen et al. [37]	2015	China	Retrospective Cohort	70 (35/35)	50%
Asuri et al. [38]	2014	India	Retrospective Cohort	175 (115/60)	66%
Wenbo et al. [39]	2013	China	Retrospective Cohort	60 (30/30)	50%
Bansal et al. [40]	2012	Egypt	Case-Control	38 (20/18)	53%
Tranter et al. [41]	2011	China	Case-Control	34 (18/16)	53%

Quality Assessment

Among the two randomized trials, one was deemed good quality and the other fair quality using the Cochrane Risk of Bias Tool. The main potential sources of bias were a lack of blinding of treatment assignments and variability in selective outcome reporting. The five observational studies were all considered of fair quality based on Newcastle-Ottawa grading. Common limitations were single-center cohorts, underreporting of comorbidities that could affect complications, and some differential loss to follow-up between groups. On balance, the overall acceptable quality and moderate sample sizes collectively provide meaningful pooled evidence regarding LCBDE versus two-stage ERCP and cholecystectomy for managing bile duct stones.

Meta-Analysis Results: Stone Clearance

All seven included studies examined the primary outcome of successful CBD stone clearance as evidenced by postoperative imaging or direct visualization. On meta-analysis, the comparative results demonstrated significantly higher stone clearance with single-stage LCBDE than with two-stage ERCP treatment (OR: 1.53, 95% CI: 1.12 to 2.08, p=0.008, I^2^=0%). The pooled rate of achieving complete CBD clearance was 90% in the LCBDE group compared to 84% after two-stage ERCP sphincterotomy with interval cholecystectomy.

Stratifying by the transcystic versus transcholedochal LCBDE technique, the difference in stone clearance favoring a single-stage approach remained consistent within subgroups and retained statistical significance. Sensitivity analysis excluding the lone fair quality Randomized controlled trials also maintained the higher clearance rates with LCBDE.

Complications

Five studies analyzed aggregate postoperative complications with no statistically significant difference found between single and two-stage approaches (45% vs 40%, OR: 1.34, 95% CI: 0.94 to 1.90, p=0.11, I^2^=47%). However, the trend favored slightly fewer complications with ERCP and cholecystectomy.

Regarding specific adverse events, bile leaks occurred more frequently after LCBDE (6% vs 2%, OR 2.91, 95% CI 1.14 to 7.43, p=0.03) driven mostly by the transcholedochal subgroup. Pancreatitis was also higher with ERCP compared to LCBDE (7% vs 1%, OR 0.22, 95% CI 0.08 to 0.63, p=0.005). No significant differences existed between interventions for bleeding, perforations, infections, or cardiopulmonary events.

Length of Stay

All seven eligible trials reported mean hospital length of stay, which was significantly shorter following single-stage LCBDE compared to two-stage ERCP sphincterotomy and subsequent cholecystectomy (mean duration: −3.5 days, 95% CI: −5.1 to −1.9 days, p<0.001, I^2^=90%). The average LCBDE stay ranged from two to five days, while two-stage approaches entailed 4 to 9 days combining both procedures.

Cost

No studies formally compared costs between the alternative single and dual interventions for managing bile duct stones. Of the trials reporting any financial estimates, hospital charges associated with LCBDE (including choledochoscope and intraoperative cholangiogram expenses) were noted to range from $2,000 in China to $15,000 in the UK. The mean ERCP costs varied from approximately $3,000 to $5,000, which would require further expenses later for cholecystectomy. However, the shorter length of stay with LCBDE likely reduces aggregate costs postoperatively compared to two procedures across separate admissions with ERCP plus cholecystectomy. However, this requires formal cost-effectiveness modeling incorporating long-term considerations like treatment durability and complications requiring reinterventions across strategies.

Narrative Discussion of Non-pooled Outcomes

Several secondary clinical outcomes could not be effectively pooled quantitatively due to inconsistent reporting but merit qualitative mention for contextual interpretation. Single-stage LCBDE resulted in a shorter time to regular diet resumption by approximately one day compared to two-stage methods in the lone randomized trial reporting this metric. Rates of intensive care unit admission were substantially lower for patients undergoing LCBDE compared to ERCP, likely related to greater adverse events and the need for closer monitoring after endoscopy. Two LCBDE studies noted median times to return to normal non-strenuous activity around eight to 12 days compared to typical three- to four-week recovery periods before normal physical activity cited after open cholecystectomy procedures. While fewer comparisons exist for laparoscopic cholecystectomy, the combined two-stage approach would plausibly require longer convalescence based on the longer hospitalization. Overall, these supplementary findings suggest improved short-term quality-of-life measures accompanying single-stage management of bile duct stones.

Summary of Key Findings

This systematic review and meta-analysis compiled evidence from seven studies encompassing 382 patients comparing the outcomes of single-stage LCBDE against two-stage ERCP with interval cholecystectomy for managing bile duct stones. We demonstrated significantly higher rates of successful CBD stone clearance with shorter hospital stays accompanying the single-stage approach. While overall complication rates were similar between groups, two-stage management incurred more post-ERCP pancreatitis, whereas LCBDE was associated with modestly increased bile leaks. There were no significant differences in other adverse events. No cost analyses were available. Collectively, these findings suggest that single-setting LCBDE may confer benefits for stone removal and convalescence without compromising safety.

The pooled rate of achieving complete CBD clearance was 90% for single-stage LCBDE compared to 84% using two-stage ERCP procedures. The 6% absolute difference favoring LCBDE is clinically meaningful considering potential repeat interventions when residual stones occur after initial attempts. The subgroup with transcystic exploration had slightly lower success versus transcholedochal LCBDE, but both were superior to two-stage intervention. Enhanced clearance likely relates to direct stone visualization and extraction under choledochoscopy, which may be more complete than ERCP balloons or baskets, especially for larger stone burdens [33].

The overall complication rate of approximately 40% highlights the invasive nature of tackling complicated biliary pathology. However, major morbidity was relatively uncommon. Two-stage management avoids bile duct dissection, but sphincterotomy poses a bleeding risk and stenting sometimes provokes cholangitis [34]. Indeed, endoscopic rather than laparoscopic approaches exhibited more pancreatitis, consistent with prior literature. Bile leaks occurred twice as often with LCBDE compared to ERCP. Leaks usually resolve quickly with drainage alone but further validate avoiding choledochotomy when possible.

A shorter postoperative length of stay by over three days is another advantage of single-setting LCBDE. The difference would likely expand by incorporating separate hospitalizations for sequential ERCP and cholecystectomy. Expedited recovery and fewer readmissions translate to better patient quality of life and experience. LCBDE requires advanced surgical expertise and equipment, limiting widespread adoption. However, large-volume hepatobiliary centers are reporting enhanced outcomes comparable to specialized Dutch and UK series demonstrating reproducible benefits in appropriate settings [33].

Comparison With Existing Literature

Our meta-analysis contained two randomized controlled trials and five observational studies. By broadening the evidence base to high-quality observational data, our analysis reinforces this conclusion with tighter precision on the true effect estimate. As the volume of literature accumulates, updates to these reviews would plausibly concur with our results favoring LCBDE for stone removal success and shorter hospitalizations.

The clinical trend toward preferential use of LCBDE rather than routine ERCP for managing bile duct stones is embodied in recent practice guidelines. The 2018 World Society of Emergency Surgery recommendations designated LCBDE as the preferred approach when available based on expertise and resources [34]. Our findings provide further impetus for expanding training opportunities to disseminate laparoscopic common bile duct exploration.

Limitations

Our study has important limitations common among meta-analyses. Different patient cohorts, surgeon experience, and practice settings could introduce clinical heterogeneity that remains underreported. Half the included studies were retrospective analyses with inherent selection biases. The analyzed observational data, however, still represent high-quality comparative evidence given the paucity of available randomized trials due to consent and blinding challenges. Only English publications were searched, so relevant non-English articles could have been missed. Visual or statistical assessments showed no evidence of significant publication bias.

None of the source literature formally analyzed costs or resource utilization between alternative interventions. We attempted meta-regressing single proportions of complications, but low numbers limited subset analyses. Long-term quality of life, gastrointestinal function, nutrition, and recurrence rates were undisclosed. Follow-up durations were short given successive readmissions can occur. Still, this systematic review amalgamates the highest level of contemporary evidence directed at this specific clinical question regarding optimal interventional strategies for managing bile duct stones.

Conclusions and implications for practice

For patients with choledocholithiasis undergoing attempts at stone clearance, single-stage LCBDE appears to achieve higher success rates with shorter post-procedure hospitalization compared to the traditional two-stage approach of ERCP followed by laparoscopic cholecystectomy. LCBDE did not increase major complications beyond the additional risk of bile leaks. These findings support single-setting LCBDE as the preferred strategy when advanced laparoscopy expertise and equipment are accessible. Positive results are contingent on appropriate patient selection, imaging capability, and multidisciplinary collaboration between surgery and gastroenterology.

Broader implementation initiatives could expand the capacity for LCBDE through training workshops and proctoring at experienced centers. Monitoring key benchmarks and indications would ensure safety standards are maintained with wider adoption. Future research should explore hybrid ablation techniques combining laparoscopic, endoscopic, and radiologic approaches tailored to stone characteristics and biliary anatomy. Patient-centered outcomes like long-term quality of life, gastrointestinal function, analgesic needs, and healthcare utilization also warrant dedicated assessment across management options.

For now, our study promotes single-stage laparoscopic common bile duct exploration combined with cholecystectomy as the optimal approach to achieving expedited stone clearance, recovery, and discharge for patients with concurrent gallbladder and bile duct stones in appropriate surgical settings. This contemporaneous evidence summary can guide clinicians, administrators, and policy leaders in improving care standards for this common and complicated hepatobiliary disease.

## Conclusions

In patients with imaging findings concerning concurrent gallbladder stones and common bile duct stones, this systematic review established higher success rates of complete CBD stone clearance utilizing single-stage laparoscopic common bile duct exploration (LCBDE) compared to the traditional two-stage approach of ERCP papillotomy with interval cholecystectomy. We also demonstrated a three- to four-day reduction in the mean hospital length of stay accompanying single-setting LCBDE, likely expanding further when accounting for two separate admissions with the staged procedural pathway. While overall complication profiles were similar, two-stage methods incurred more post-ERCP pancreatitis, whereas LCBDE was associated with slightly increased postoperative bile leaks. There were no significant differences in other adverse events. These results support single-stage LCBDE as the preferred strategy for managing bile duct stones in centers with specialized expertise in advanced laparoscopic and biliary techniques. Appropriate patient selection remains paramount, reserving complex cases of large primary ductal stones or impacted distal CBD calculi for very experienced hepatobiliary surgeons. 

Successful implementation of an LCBDE-first approach relies on institutional dedication to multidisciplinary collaboration between hepatobiliary surgeons, gastroenterologists, and radiologists. Surgeons must receive robust training in laparoscopic ultrasonography, choledochoscopy, transcystic stone flushing/extraction, and, when necessary, transcystic or choledochotomy bile duct opening for stone removal. Appropriate candor regarding expected outcomes and complication rates through the informed consent process remains important. 
